# A trait-based trade-off between growth and mortality: evidence from 15 tropical tree species using size-specific relative growth rates

**DOI:** 10.1002/ece3.1186

**Published:** 2014-09-05

**Authors:** Christopher D Philipson, Daisy H Dent, Michael J O’Brien, Juliette Chamagne, Dzaeman Dzulkifli, Reuben Nilus, Sam Philips, Glen Reynolds, Philippe Saner, Andy Hector

**Affiliations:** 1 Mountain Ecosystems, WSL Institute for Snow and Avalanche Research, SLFFlüelastrasse 11, CH-7260, Davos Dorf, Switzerland; 2 Institute of Evolutionary Biology and Environmental Studies, University of ZurichZurich, Switzerland; 3 Biological and Environmental Sciences, University of StirlingStirling, UK; 4 Smithsonian Tropical Research InstituteApartado, Postal 0843-03092, Balboa, Panama; 5 Forest Research CentreSepilok, Sandakan, Sabah, Malaysia; 6 Kasanka National ParkZambia, Central Province, Zambia; 7 The Royal Society South-East Asian Rainforest Research Programme, Danum Valley Field CentreSabah, Malaysia; 8 Department of Plant Sciences, University of OxfordSouth Parks Road, Oxford, OX1 3RB, UK

**Keywords:** Dipterocarpaceae, functional traits, light, nonlinear growth, plant development and life-history traits, SGR, survival, tropical lowland forest, wood density

## Abstract

A life-history trade-off between low mortality in the dark and rapid growth in the light is one of the most widely accepted mechanisms underlying plant ecological strategies in tropical forests. Differences in plant functional traits are thought to underlie these distinct ecological strategies; however, very few studies have shown relationships between functional traits and demographic rates within a functional group. We present 8 years of growth and mortality data from saplings of 15 species of Dipterocarpaceae planted into logged-over forest in Malaysian Borneo, and the relationships between these demographic rates and four key functional traits: wood density, specific leaf area (SLA), seed mass, and leaf C:N ratio. Species-specific differences in growth rates were separated from seedling size effects by fitting nonlinear mixed-effects models, to repeated measurements taken on individuals at multiple time points. Mortality data were analyzed using binary logistic regressions in a mixed-effects models framework. Growth increased and mortality decreased with increasing light availability. Species differed in both their growth and mortality rates, yet there was little evidence for a statistical interaction between species and light for either response. There was a positive relationship between growth rate and the predicted probability of mortality regardless of light environment, suggesting that this relationship may be driven by a general trade-off between traits that maximize growth and traits that minimize mortality, rather than through differential species responses to light. Our results indicate that wood density is an important trait that indicates both the ability of species to grow and resistance to mortality, but no other trait was correlated with either growth or mortality. Therefore, the growth mortality trade-off among species of dipterocarp appears to be general in being independent of species crossovers in performance in different light environments.

## Introduction

Light is generally accepted to be the most limiting resource in tropical rain forests (Whitmore and Brown 1996) and has long been hypothesized to be important in the maintenance of tree species diversity (Denslow 1987). Two mechanisms have been proposed to explain how differences in species-specific responses to light availability may maintain species diversity in tropical forests. First, species that grow well in one light environment have relatively lower growth rates in other light environments (Sack and Grubb 2001, 2003). Second, a trade-off may exist in plant functional traits that result in either low mortality in the shade or rapid growth in high light, but not both (Kitajima and Bolker 2003). However, the relative importance of these two mechanisms continues to be debated (Baraloto et al. 2005; Kitajima and Poorter 2008; Dent and Burslem 2009; Kunstler et al. 2009).

The first mechanism involves partitioning of the habitat with each species performing best (in terms of growth) in a particular light environment. This light partitioning is a result of species responding differently to the light gradient (i.e. a statistical interaction between species and light) and crossing-over in their rank performance as light availability changes. However, there is much debate over whether the magnitude of differential species responses to light is sufficient to generate rank crossovers (Sack and Grubb 2001, 2003; Kitajima and Bolker 2003). Many studies present data that illustrate species–light interactions, that result in crossovers in rank performance and therefore show some support for species specialization to distinct light environments (Sack and Grubb 2001, 2003; Baltzer and Thomas 2007; Philipson et al. 2012). However, there is very limited evidence for a strong trade-off between growth in high-light versus growth in the low-light environments, with the general consensus being that this is unlikely to occur (Sack and Grubb 2001; Philipson et al. 2012). Furthermore, Kitajima and Bolker (2003) present a detailed analysis showing that crossovers in rank are not statistically supported and rank reversals in performance among light treatments rarely occur. Instead, they argue that low mortality in the shade trades off with rapid growth in high light. This trade-off is a widely accepted mechanism underlying plant ecological strategies in tropical forests (Grubb 1977; Kitajima and Poorter 2008; Wright et al. 2010).

Thus, light has been hypothesized to contribute to species coexistence either via species-specific growth responses to a light gradient or via a trade-off between growth in the light and mortality in the shade. These two mechanisms are not necessarily mutually exclusive – species may crossover in growth responses along a light gradient and also species that grow fast in the light, may have increased mortality in the shade. Both mechanisms underpin a gradient in ecological strategy ranging from light-demanding pioneers at one end of the spectrum to highly shade-tolerant canopy trees at the other. Pioneers tend to have high germination rates and maximum growth rates in open disturbed sites, but high mortality in the shade (Swaine and Whitmore 1988). In contrast, shade-tolerant canopy trees tend to have high germination rates and low mortality in deep shade, yet their growth rates are constrained. These disparate ecological strategies, which govern regeneration dynamics, are defined by functional traits and can be illustrated by trade-offs that occur when the functional traits that maximize the fitness of plants in one environment are below-optimal in another environment (Dalling and Burslem 2005; Poorter et al. 2008).

Functional traits such as seed mass, wood density, specific leaf area (SLA), and leaf nutrient concentrations underlie differences in these ecological strategies and are correlated with growth and mortality (Ackerly 2003; Paz and Martinez-Ramos 2003; Poorter and Bongers 2006; Poorter et al. 2008; Wright et al. 2010). There is substantial evidence that functional traits determine the position of a species along an axis ranging from species that maximize resource-capture and have rapid growth rates at one end of the spectrum to species adapted to resource-conservation and with low mortality at the other end (Kitajima and Poorter 2008, 2010; Wright et al. 2010). For example, it is generally the case that pioneer trees produce large numbers of small seeds and have low wood density and thin, poorly defended leaves with high nutrient concentrations while shade-tolerant species have the opposite suite of traits; small numbers of large seeds, high wood density etc. (Whitmore 1998). However, the relative importance of particular traits and the relationships among traits and growth and mortality rates, particularly within functional groups is poorly understood (Larjavaara and Muller-Landau 2010; Wright et al. 2010).

These categories of pioneers versus shade-tolerant climax species are established extremes in ecological strategy along the continuum of functional groups (Kariuki et al. 2006), and yet within these functional groups, there is also high species diversity. Some of the most diverse tropical forests found are on the island of Borneo, where 267 species of trees from the family Dipterocarpaceae dominate the forest canopy (Ashton 1982). Dipterocarps are shade-tolerant canopy trees and represent just one functional group, within which there is a gradient in ecological strategy including a range of mortality and growth rates (Becker et al. 1998; Philipson et al. 2012). We propose that trade-offs in functional traits may explain the variation in growth and mortality rates and contribute to the maintenance of species diversity within this functional group.

We present 8 years of growth and mortality data for 15 species of Dipterocarpaceae saplings planted under heterogeneous canopy cover within an experimental area of selectively logged forest in Malaysian Borneo (The Sabah Biodiversity Experiment; (Hector et al. 2011)). We investigate the relationship between mortality growth rates along a light gradient and determine whether functional traits underpin a trade-off between growth and mortality.

Specifically, we show the following:

1. There is very limited evidence that species respond differently to variation in light conditions in terms of both growth and mortality rates; all show faster growth rates and declining mortality with increasing light.
2. There is a clear trade-off between mortality and growth rates in this phylogenetically and functionally constrained group of shade-tolerant trees despite the lack of clear differences in species-specific responses to the light gradient.
3. Of the plant functional traits tested, only wood density was correlated with growth and mortality rates.

## Materials and Methods

### Study site

The Sabah Biodiversity Experiment (SBE) is located in the Malua Forest Reserve in the eastern region of the Malaysian state of Sabah, Northern Borneo, see Hector et al. (2011) for full details. The Malua forest reserve is embedded within the Yayasan Sabah Forest Management Area (YSFMA); a 1 million hectare forest concession that includes Danum Valley Conservation Area, extensive areas of forest under management for timber production (approx. 750,000 hectares), and two of SE Asia’s largest forest rehabilitation projects (the Sabah Biodiversity Experiment within Malua Forest Reserve, SBE; and the INnoprise – Face foundation Rainforest Rehabilitation PROject, INFAPRO). The region has no distinct seasons with an annual rainfall of around 3000 mm per year (Saner et al. 2011; O’Brien 2013). Temperatures recorded at Danum Valley Field Center (DVFC) were found to be typical of a wet equatorial climate with mean daily temperatures of 26.8°C (Clarke and Walsh 2006).

The seedlings in this study were planted into lowland mixed dipterocarp forest that was logged in the late 1980s. The trees targeted during the logging operation were mostly dipterocarps, which are thus depauperate in the remaining forest. The saplings included in this analysis are part of the SBE forest rehabilitation project, which specifically investigates the effect of altering the diversity of dipterocarp trees by enrichment planting (Hector et al. 2011). The INFAPRO enrichment-planting project is one of the most extensive in SE Asia, and the SBE follows the planting techniques of INFAPRO’s enrichment-planting system as closely as possible, with a view to making the most relevant recommendations for the regions humid forests.

### Seedling sources and planting

Seedlings were sourced from INFAPRO. Seeds were collected from the surrounding forest within the YSFMA. Seeds were then stored under wet jute sacks until germination then sown into polyethylene pots (7 × 23 cm) filled with shredded locally collected topsoil. Seedlings were grown under shade-cloth in 10% ambient light and watered twice daily for a minimum of 3 months before being transplanted into the experimental SBE plots (Hector et al. 2011). All the saplings included in this study are from six high diversity treatment plots (Hector et al. 2011). The average seedling diameter just before planting was 4.12 mm, (range: 2.74–8.25 mm). The average number of individuals with more than three size measures required for the growth analysis was 68 (range: 12–132). Each experimental plot (200 × 200 m) consists of 20 planting lines separated by 10 m. Each 200 × 2 m planting line was created by manually clearing the understory of seedlings, shrubs, bamboo, and lianas. Seedlings were planted every 3 m along the center of each planting line, unless there was a physical obstruction (river, rocky outcrop, etc.). Planting was carried out throughout 2002 and 2003. Planting lines were cleared and maintained when required; up to twice annually. The SBE planting methods are described in greater detail in (Hector et al. 2011). Six plots to the west of block one, each including all species, were selected for intensive measurements of diameter growth and mortality.

### Study species

The species selected for the SBE were restricted to those which (i) had sufficient availability at the time of planting in 2002 and 2003, (iii) were broadly representative of the species composition of the study site, and (iii) included a range of commonly occurring traits (see Table 1). Fifteen closely related species within the family Dipterocarpaceae were selected. Although all species are shade-tolerant, late-successional canopy trees, they were selected specifically to span the range of shade-tolerance and timber quality within this group: *Shorea johorensis* Foxw., *Shorea gibbosa* Brandis., *Shorea argentifolia* Sym., *Shorea faguetiana* Heim., *Shorea leprosula* Miq., *Shorea macrophylla* Ashton, *Shorea macroptera* King, *Shorea ovalis* Korth., *Shorea parvifolia* Dyer. *Shorea beccariana* Bruck, *Parashorea malaanonan* (Blanco) Merr., *Parashorea tomentella* (Blanco) Merr., *Hopea sangal* Korth., *Dryobalanops lanceolata* Burck, *Dipterocarpus conformis* Slooten. One species present in the SBE experiment, *Hopea ferruginea* Parij, suffered extremely high mortality immediately after planting and before the first data were collected, resulting in insufficient replication for analysis and has therefore been omitted from this study. The abundance and distribution of these species is described in Table 1.

**Table 1:** Abundance and distribution of the Dipterocarp species used in this study. Data on abundance and distribution were obtained from Meijer & Wood (1964), Ashton (1982) and Newman *et al*. (1999 & 1998)

Genus	Species	Abundance and distribution
*Dryobalanops*	*lanceolata*	Widespread on fertile soils, abundant on undulating land, to 700 m
*Dipterocarpus*	*conformis*	Rare, hill dipterocarp forest, clay-rich soils, below 800 m
*Hopea*	*sangal*	Often on or near river banks in low country and to 500 m
*Parashorea*	*tomentella*	Common on flat to rolling hills below 200 m
*Parashorea*	*malaanonan*	Local on clay-rich soil, rarely on riverbanks, on ridges in mountains to 1350 m
*Shorea*	*beccariana*	Common, lowlands, and dry ridges to 1350 m
*Shorea*	*argentifolia*	Locally frequent on ridges, hillsides, and valleys, usually below 600 m
*Shorea*	*faguetiana*	Low hills and particularly ridge tops at 150–700 m, occasionally to 1000 m
*Shorea*	*gibbosa*	Locally common on the most fertile clay-loam soils on undulating land and river banks to 650 m
*Shorea*	*johorensis*	Very common on well-drained fertile soils below 600 m
*Shorea*	*leprosula*	Widespread, fast-growing emergent, common below 700 m
*Shorea*	*macroptera*	Common, sandy clay soils on low hills to 600 m
*Shorea*	*ovalis*	Scattered, usually in moist places in valleys and low-lying ground, occasionally ultrabasics, to 500 m
*Shorea*	*parvifolia*	Perhaps the commonest dipterocarp, on clay soils on hills below 800 m
*Shorea*	*macrophylla*	Locally abundant on periodically flooded alluvium and near rivers, uncommon on hillsides, below 600 m

### Seedling measurements

**Figure 1:**
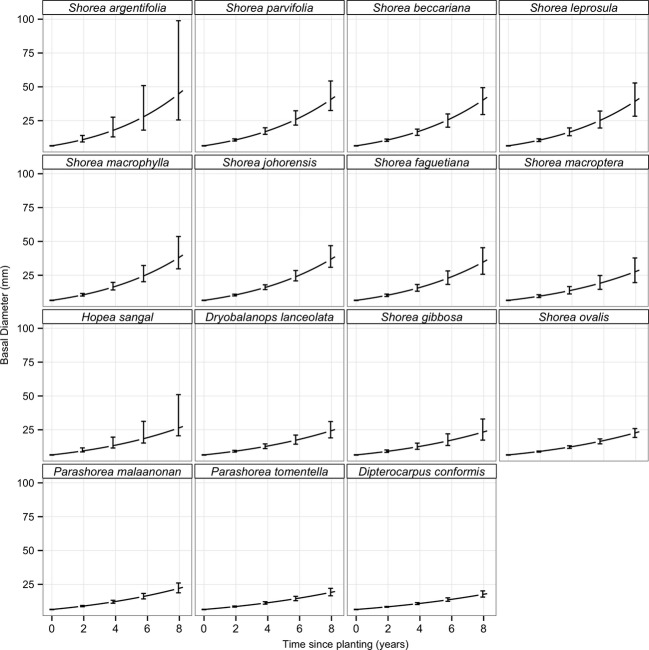
Modeled basal diameter through time for saplings of 15 Dipterocarp species. Panels are ordered by the fastest growing species (*Shorea argentifolia)* in the top left to the slowest growing species (*Dipterocarpus conformis)* in the bottom right. The diameter growth model explained a large amount of the variation with a Pseudo *R*^2^ = 0.986. Predictions are based on individual seedlings of a mean size (6.4 mm diameter) and experiencing a mean canopy openness (4.5%), and are therefore not confounded by size differences between species. The error bars are 95% confidence intervals calculated by sampling the parameter estimates 1000 times and fitting the power-law through time for each of the 1000 runs.

All seedlings were measured and censused for mortality on average 576 days after planting. Seedlings were censused a further six times from 2004–2011, after approximately, 815, 1166, 1474, 2885, 3214, and 3214 days after planting. At each census, seedling diameter was measured 2 cm from the soil surface and at breast height (1.3 m) if plants were sufficiently tall. Light conditions were assessed at every census using spherical densiometers directly above each seedling (Lemmon 1956)**.** Canopy openness across the study site varied between 0.5% and 25%, representing the full range present in the primary forest. The smallest variation in canopy openness above an individual seedling ranged from 1–15% for *S. argentifolia* while the largest variation was 0.5–26% for *H. sangal* (Fig. 1).

### Trait measurements and estimates

Functional trait data from four experiments conducted at the site were combined to generate a database of leaf and whole plant traits (Philipson 2009; Philipson et al. 2012; O’Brien 2013). Linear mixed-effects models were used to estimate mean functional traits. Random intercepts were added for species, experiment, and any other design or treatment variables key to any of the datasets. Estimates for the traits where extracted using only the grand mean and species random effects.

#### Leaf traits

In all four experiments, all leaves were collected from all harvested plants, 3285 in total, providing leaves for estimating specific leaf area (SLA). Leaf area was estimated at the time of harvest by photographing all leaves and calculating the area using imageJ software (Abràmoff et al. 2004). Leaves were then dried at 60°C to constant mass and weighed without petioles. Leaf area and dry mass were used to generate SLA values. The leaves of the final harvest for two experiments (Philipson 2009; Philipson et al. 2012) were dried, ground, and the total nitrogen was extracted using the Kjeldahl method. Organic carbon was estimated following Walkley and Black (1934).

#### Whole plant traits

Wood density estimates were generated from the saplings from all three light environments in the shade-house experiment described in Philipson et al. (2012). These seedlings used were the same planting material from INFAPRO nursery. Specifically, wood density was measured for all saplings at the final harvest, after almost 2 years of growth, using approximately 30 cm of the lower stem and following the protocols outlined by Chave (2005). Seeds were collected during a masting event in 2010 for two experiments conducted at the site (O’Brien et al. 2013, 2014). A total of 1887 seeds were collected, (range: 49–137 per species), oven-dried, and individually weighed.

### Data analysis

Recent studies have shown that accounting for size differences among species can change our interpretation of growth rate data (Turnbull et al. 2008, 2012; Hautier et al. 2010; Rees et al. 2010; Philipson et al. 2012).

Put simply, because relative growth rate (RGR) generally declines as organisms grow, comparisons of RGR calculated for species of different sizes risk attributing differences in growth rate due to size to intrinsic species differences.

Therefore, we allowed for the estimation of growth rates at a specific size by fitting a curve to multiple diameter measurements through time. Growth was modeled as a power-law, following the method described in detail in Philipson et al. (2012), where the absolute growth rate is given by

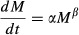
(1)where *α* is a growth coefficient, *β* is the scaling exponent, and *M* is plant size. Equation 1 has the following analytical solution when *β* ≠ 1:

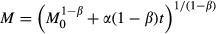
(2)where *M*_*0*_ is the initial size (more details of the derivation are provided in Philipson et al. 2012). A *β* of zero would indicate constant linear growth not dependent on size, whereas a *β* of one would indicate exponential growth, with no slowing of growth with size. Note that equation 1 has a change of form of the solution when *β* = 1 (Philipson et al. 2012), but in this study, *β* was lower than 1 so this did not apply. Equation 2 was fitted to diameter data by estimating *M*_*0,*_ *α* and *β* using nonlinear mixed-effects models. In order to compare growth rates to mortality rates among species at a common size, we then extracted the parameters from the fitted model and calculated a size-specific relative growth rate (SGR) as:

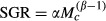
(3)where *M*_*c*_ is a common reference size. Because species shared a single value of the scaling exponent *β* (see results), differences in SGR among species and light in our results are solely due to differences in the growth coefficient, *α,* and relative rankings do not depend on the choice of the reference size. Note that SGR is still relative growth rate (RGR); the difference is that SGR is an instantaneous RGR at a common reference size, whereas conventional RGR calculations are averages over the growth interval and implicitly assume growth is log-linear (as it is calculated on the log size scale).

Canopy openness was measured at every census interval. The model that gave the best fit to the data used an average of the canopy openness measures. Note that when saplings were substantially taller than the height at which densiometer measurements were recorded, the estimate reflects the light environment created by the tree rather than experienced by the tree. For this reason, we tested various models: the model that fitted best used the average canopy openness until saplings exceeded 160 cm in height, after which the same average value was used.

All analyses were carried out in R 2.15.1 (R Core Team 2012). Growth was analyzed using nonlinear mixed-effects models with the nlme() function in version 3.1–104 of the *nlme* package (Pinheiro et al. 2012). The models were parameterized with the substantial dataset of over six thousand diameter measurements on more than a thousand seedlings. We followed the detailed advice provided in Pinheiro & Bates (2000) for model fitting and simplification. Individual seedling identity was fitted as a random effect so that the full model includes an effect of seedling identity on all three parameters. Further simplification of the random effects was attempted, but not possible, that is all three parameters were allowed to vary between individuals. We identified the most parsimonious model (fitted using maximum likelihood) based on minimizing Schwarz’s (1978) Bayesian Information Criteria (BIC). Models with a BIC of more than two points lower were considered better. Species (a factor with a level for each species) and canopy openness (continuous) were treated as fixed effects. We fitted both canopy openness and log canopy openness, but models with log canopy openness were always preferred (as judged using BIC). In the most parsimonious model, *α* varied with species and with the log of average canopy openness while there was a common value of *β* and *M*_*0*_ for all species and canopy openness. The diameter measures were not log-transformed, and therefore, the initial residuals were strongly heteroscedastic. To account for heteroscedasticity, we allowed a unique variance for each species using the *varIdent* function with the *weights* argument in *nlme*. Models were compared when they were fitted with maximum likelihood (ML), but all parameter estimates were taken from the final models fitted using restricted maximum likelihood (REML) as recommended by Pinheiro & Bates (2000). Mixed-effects models do not report a traditional *R*^2^; therefore, pseudo-*R*^2^ was calculated using the squared correlation of fitted and observed values. All models were checked using both plots of the predictions against the raw data and plots of the residuals against the fitted values. Models that had patterns in the residuals, or produced very poor fits to the data were discarded. All models that had sensible predictions and residuals that met with model assumptions were compared using BIC.

Mortality data were analyzed using generalized linear mixed-effects models with the glmer() function in version 0.999999-0 of the lme4 package (Bates et al. 2013). We predicted the probability of mortality of seedlings given their diameter, species and the canopy openness assuming a binomial error distribution and a complementary log-log link. The intercepts were allowed to vary for both individual seedling and measurement in time as normally distributed random effects (random intercept model). Irregularly spaced census intervals necessitated an offset using log-transformed time since the previous census which produces parameter estimates scaled in units of years rather than census intervals (Barker and Press 1997; Egli and Schmid 2001; Bagchi et al. 2011).

A matrix of Pearson’s correlation coefficients was calculated for the species-specific estimates of the growth and mortality measures and four key functional traits (wood density, SLA, leaf C:N ratio and seed mass). Significant correlations were then plotted, and relationships were further investigated using Standardized Major axis regression using the sma() function in version 3.2.6 of the smatr library (Warton et al. 2011).

## Results

### Growth

Growth varied among species; *Shorea argentifolia,* the fastest growing species, had a growth coefficient intercept, *α*, of 0.000908 mm mm^−1^ day^−1^ (95% CI: 0.000583–0.00142), while for the slowest growing species *Dipterocarpus conformis*, the growth coefficient intercept was *α* = 0.000427 mm mm^−1^ day^−1^ (95% CI: 0.000328–0.000555). Diameter growth, *α*, increased linearly with log canopy openness with a slope of 0.092 (95% CI: 0.061–0.123). Growth rate increased with increasing canopy openness, but there was no statistical interaction between species and log canopy openness (ΔBIC On removing interaction = 99.20), which indicates no clear difference between species in their growth responses to light. Species did not exhibit exponential growth as indicated by the common scaling exponent, *β*, which for all species and light treatments was estimated at 0.86 (95% CI: 0.77–0.94; Fig. 1); a *β* of zero indicates linear growth, whereas a *β* of one indicates exponential growth. This suggests that increased self-shading, allocation to structural tissue, etc. result in slower growth rates for larger individuals as expected. The diameter growth model explained a large amount of the variation with a Pseudo-*R*^2^ of 0.986. The high value must be interpreted remembering that the model includes random effects for both among and within species variation (individual level random effects accounts for within species variation).

### Mortality

**Figure 2:**
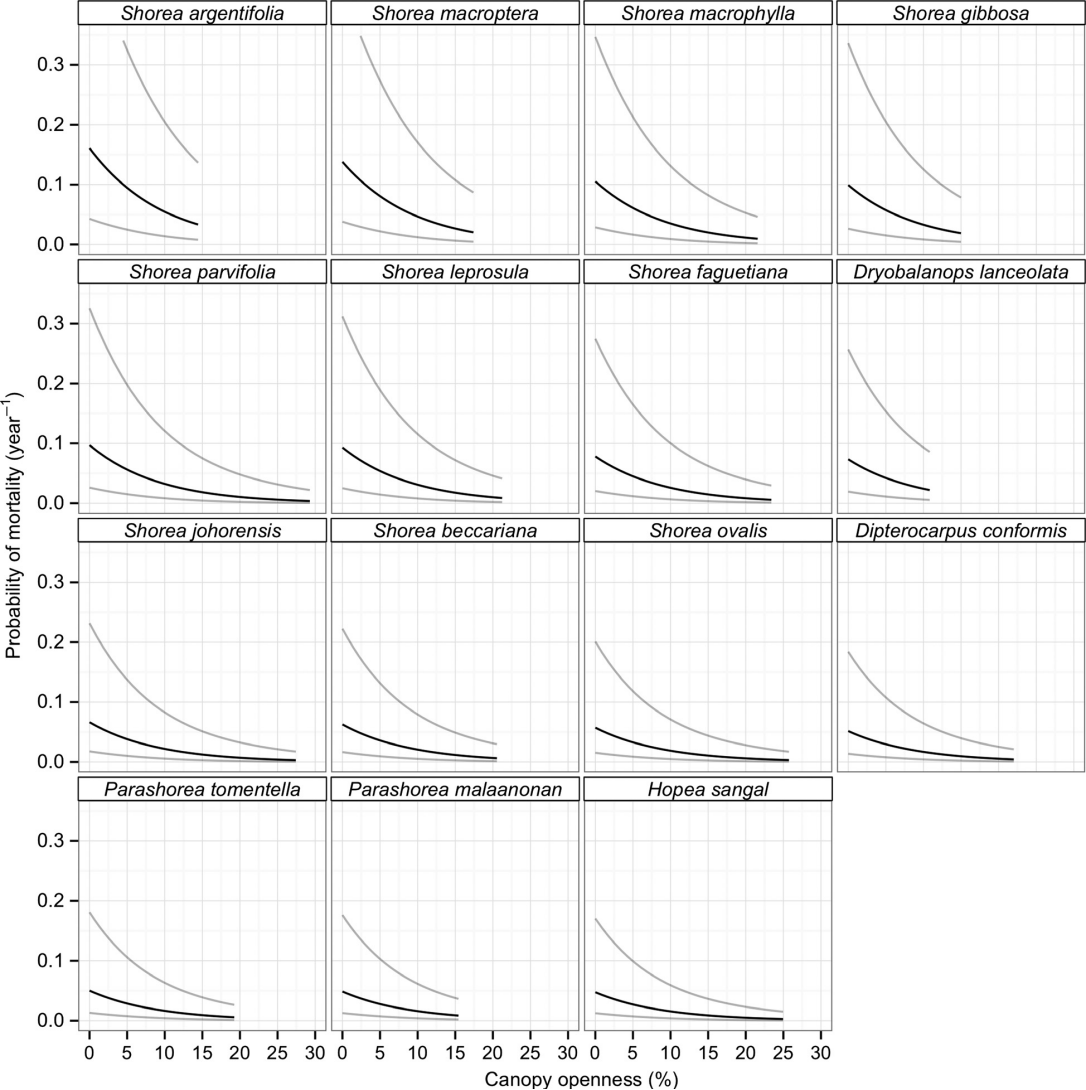
Probability of mortality estimated at a common size in response to percentage of canopy openness. Panels are ordered by species mortality with the species with the highest mortality in the shade in the top left (*Shorea argentifolia)* and the species with the lowest mortality in the bottom right (*Hopea sangal)*. Estimates are only plotted for the range of canopy openness observed in the dataset for that species. These predictions were generated using a model with no species light interaction, so the response to light is the same for each species. Thus, the curve represents a section of a logistic curve, and only the intercept and the range of data determine species differences.

Probability of mortality decreased with initial diameter of seedlings, regardless of species (*β*_inital diameter_ = −0.01; 95% CI: −0.03-0.01). After initial size differences were taken into account, there was an effect of both light and species on seedling mortality. The probability of mortality decreased with increasing canopy openness (*β*_canopy openness_ = −0.11; 95% CI: −0.16–0.07). And species had different intercepts in their probability of mortality; *Shorea argentifolia* had the highest mortality rate of 0.182 year^−1^ (95% CI: 0.050–0.514) while *Hopea sangal* had the lowest of 0.054 year^−1^ (95% CI: 0.014–0.185; Fig. 2)**.** However, there was no statistical interaction between species and canopy openness (ΔBIC on removing interaction = −91.25), indicating minimal evidence for differences among species mortality in response to light.

### Growth mortality trade-off

**Figure 3:**
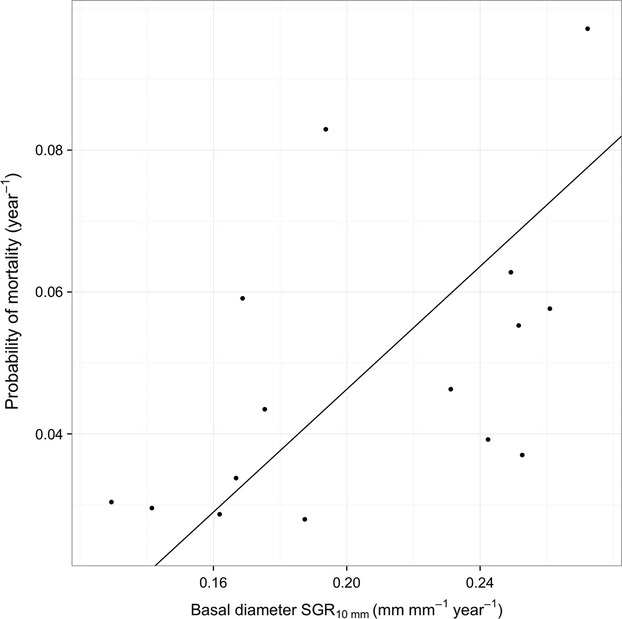
Mean probability of mortality plotted against growth rate of basal diameter (growth and mortality rates are calculated for 10-mm-diameter seedlings) for 15 Dipterocarp species. Line indicates Standardized Major Axis regression. *R*^2^ = 0.30, *P* = 0.036.

All species responded to light in a similar way in terms of growth and mortality (Figs. 1 and 2). Growth was positively correlated with mortality regardless of canopy openness. An increase in diameter growth rate of 0.1 mm mm^−1^ year^−1^ caused a 4.3% increase in probability of mortality year^−1^ (*β*_diameter SGR_ = 0.433 mm mm^−1^·year^−1^; 95% CI: 0.27–0.70; Table 2 and Fig. 3). The fact that both growth and mortality differ among species but without interactions between species and light, indicates that a general growth mortality trade-off is evident across all light environments. This suggests that the trade-off is driven by intrinsic species differences rather than differential species responses to light. *Shorea argentifolia,* for example, exhibited the highest probability of mortality per year and the highest growth rate. In contrast, *Parashorea tomentella* consistently had one of the lowest growth and mortality rates, regardless of canopy openness (Fig. 1 and 2).

**Table 2:** Pearson’s correlation coefficients for all pairwise combinations of growth and mortality rates and four functional traits. Significant correlations are indicated in bold and with one asterisk indicating significance at <0.05, and two asterisks indicating significance at <0.01

	Wood density	Seed mass	Leaf C:N	SLA	SGR
Wood density					
Seed Mass	−0.33				
Leaf C:N	0.49	0.27			
SLA	0.34	−0.41	−0.39		
SGR	−0.74**	0.14	−0.50	−0.17	
Mortality	−0.52*	0.10	−0.13	−0.16	0.54*

### Correlations among functional traits and growth and mortality rates

**Figure 4:**
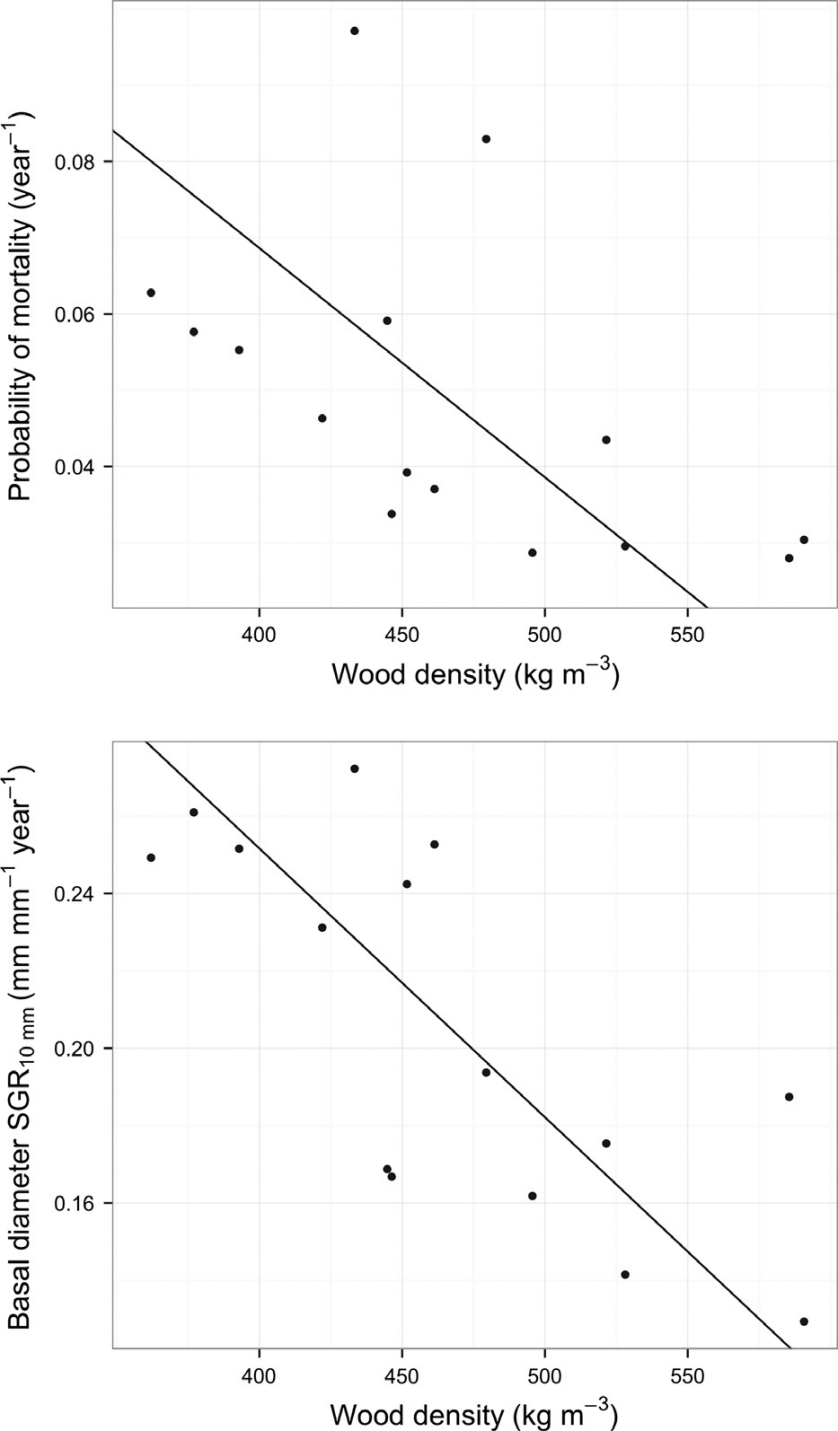
The relationships between species wood density and mean annual probability of mortality (*R*^2^ = 0.27, *P* < 0.047; top panel), and species wood density and mean basal diameter growth rate (*R*^2^ = 0.55, *P* < 0.001; bottom panel; growth, and mortality rates are calculated for 10-mm-diameter seedlings) for 15 Dipterocarp species. Lines indicate Standardized Major Axis regression.

Wood density was negatively correlated with growth and mortality (Table 2, Fig. 4). No other functional traits were significantly correlated with growth and mortality rates, or with any other functional trait (Table 2). The dataset includes only 15 species and so has limited power for predicting these bivariate relationships, nevertheless there are some correlations that are not significant and yet more than 40% of the variance was explained by the relationship. For example, leaf C:N was negatively correlated with SGR and SLA and positively correlated with wood density. These correlations were not statistically significant at the level of *P* < 0.05 with our sample sizes but may still be biologically meaningful. Seed mass and SLA were not correlated with either growth or mortality rates, but were negatively correlated with each other.

## Discussion

We found that growth increased with light for all species but that each species had different intrinsic growth rates. Interestingly, we found no evidence that species grew differently in response to light; species either grew rapidly or slowly relative to the rest regardless of light environment. Additionally, mortality decreased with increasing light, and species had different intrinsic mortality rates. Species did not respond differently to light, but species that showed high mortality in the shade also had high mortality in higher light conditions. Species that had high growth rates had high mortality rates indicating a strong trade-off between growth and mortality. Wood density was the only trait correlated with both growth and mortality, indicating that wood density could be a useful predictor of growth and mortality rates.

### Growth

Growth of all species responded positively to increasing light. There is no evidence that species responded differently to light but instead some species simply out-performed others under all light conditions (Fig. 1). This result is in contrast to many studies, which have reported variation species growth responses to light (Sack and Grubb 2001, 2003; Baltzer and Thomas 2007; Philipson et al. 2012). However, many of the studies that show crossovers in rank among some species, come from shade-house studies (Sack and Grubb 2001, 2003; Philipson et al. 2012). The data from this field-based study support no crossovers at all – rather a complete positive correlation between growth in all light environments. The apparently contradictory results may be due, in part, to the controlled conditions of shade-houses, which tend to allow greater sensitivity in detecting differences in growth rates. In addition, some of these crossovers may be due to size effects – which we have controlled for in our study. Moreover, there have been strong arguments that studies reporting crossovers are not supported statistically (Kitajima and Bolker 2003; Kitajima and Poorter 2008). The result that sapling growth rates in our field study showed no partitioning for light supports the argument that crossovers in growth rates are not statistically supported.

Our results were supported by a study by Bloor and Grubb (2003) that investigated seedling growth rates of 15 shade-tolerant tropical trees in Australia. They showed a strong positive correlation between growth rates in high and low light, and only a very limited number of crossovers in rank growth performance. Most studies that have tried to quantify the growth response of tropical trees to light have focused on a small number of species at the seedling or sapling stage (Poorter 1999; Dalling et al. 2004). However, Rüger et al. (2011a) used a hierarchical Bayesian approach to disentangle the effects of light and size on growth of a large number of tree species in Panama. Their results indicate that growth in low- and high-light environments were highly correlated across species. Furthermore, they found little evidence for light gradient partitioning in terms of growth rates among species. In combination, these studies support our findings that within a phylogenetically constrained functional group, there is minimal evidence that species specialize on particular light conditions. Instead, a single growth hierarchy exists across all light environments, suggesting a general trade-off with between growth and mortality among closely related species.

### Mortality

Mortality rates differed substantially among species, and across all species mortality rates consistently decreased with increasing canopy openness (Fig. 2). The response to light was the same for all species. This reflects the patterns seen in growth responses to light (i.e., that species appear to have intrinsic differences in their mortality rates rather than differential responses to light). Bloor and Grubb (2003) also found no interaction among species and light for mortality in shade-tolerant species. Kunstler et al. (2009) accounted for size differences in their mortality models and found a strong effect of seedling size on the interaction between light availability and species identity (i.e., smaller seedlings had a stronger light-species interaction than larger ones). Therefore, the large size of our saplings may explain the lack of a species–light interaction (Kunstler et al. 2009). Although our results cannot elucidate the potential importance of ontogenic shifts in species rank crossovers, it is clearly the next step to understanding the complex development of understorey seedling communities (Baraloto et al. 2005).

### Growth mortality trade-off

There was a positive relationship between mean growth rate and mean mortality rate (Fig. 3). A trade-off between growth in high light and mortality in low light has been reported in many studies and is a recognized process in tropical forests (Kitajima 1994; Davies 2001; Dalling and Hubbell 2002; Kitajima and Bolker 2003; Poorter and Arets 2003; Baraloto et al. 2005; Dent and Burslem 2009). The trade-off between growth in high light and mortality in low light has previously been presented as dependent on species–specific interactions with light, such that some species have high growth rates in the light and increased mortality in the shade, rather than consistently high mean growth rates and high mortality per se (Baraloto et al. 2005; Dent and Burslem 2009). However, there was no evidence for a statistical interaction between species and light for either growth or mortality in our analysis. The growth mortality trade-off we report is therefore evident in all light environments (Fig. 3) and not dependent on species crossing-over (i.e., the species all respond similarly to light). A limited number of other studies have also shown a trade-off in growth and mortality rates in specific light environments rather than only for the typical extremes. For example, Kitajima (1994) reported a trade-off between growth and mortality in low light for 15 tree species from Barro Colorado Island, Panama, and Wright et al. (2010) reported a growth mortality trade-off for average growth rates and overall mortality rates across 103 woody plant species from the same site. However, Wright et al. (2010) noted that the trade-off was strongest when growth rates of the fastest growing individuals and mortality rates of the slowest growing were evaluated. Our results suggest that it is intrinsic differences in species mortality or growth rates that explain this trade-off rather than plastic responses, drawing focus to differences in species traits, rather than to their response to the environment.

Trade-offs purely in fitness components involve species with lower mortality exhibiting lower maximum relative growth rates (Latham 1992; Kitajima and Bolker 2003; Baraloto et al. 2005). However, the more commonly observed trade-off between high mortality in low light and rapid growth in high light illustrates an interaction between fitness component trade-offs and microhabitat trade-offs, where fitness component trade-offs are only seen when microhabitat extremes are compared (Baraloto et al. 2005). This can be generalized as a trade-off between low mortality at low resource availability versus high growth at high resource availability which then promotes coexistence in heterogeneous environments (Wright et al. 2010). In support of this, studies have reported that availability of below-ground resources influences the strength of the trade-off between mortality in low light and rapid growth in high light with maximum growth trading off against mortality in the most stressful environment (for example low light and low soil nutrients; Baraloto et al. 2005; Dent and Burslem 2009). In contrast, our results indicate a fitness component trade-off between growth and mortality in all light environments, independent of microhabitat trade-offs.

This unique result may be due to a number of factors including the more stressful environment of the logged forest (such as higher temperature, drier soil, and lower nutrients); the extended time frame of this study; the fact that it was conducted in situ rather than in a controlled environment; our use of conservative mixed-effects models; the method or estimating growth and mortality independent of size effects; and the fact that we used only closely related tree species from within the shade-tolerant functional group. In support of the importance of long-term studies, Sack and Grubb (2001) examined a number of studies focusing on crossovers in species growth performance and illustrate that short-term studies do not adequately represent the processes of long-term natural establishment. Longer time periods can dilute the effect of differences in initial size – explaining contradictory results seen in the literature. We followed the growth and mortality of saplings for 8 years, representing a substantial fraction of the establishment phase. The importance of controlling for size differences in analyzing mortality data was highlighted by both Kunstler et al. (2009) and Rüger et al. (2011b) – and this may also account for some of the differences between previously published studies and the results that we present here.

### Wood density

Physical damage to seedlings from tree falls and herbivory may be important in maintaining the growth and mortality trade-off in forest environments (King et al. 2006b; Paine et al. 2012). Seedlings can be damaged in both forest gaps and understory sites and so having defense traits, particularly tough wood (King et al. 2006a), well-defended leaves (Coley and Barone 1996; Alvarez-Clare and Kitajima 2007) and carbohydrate storage (Myers and Kitajima 2007; O’Brien et al. 2014), may decrease the likelihood of mortality in all forest environments. We found that both growth and mortality were strongly negatively correlated with wood density. This result is similar to that reported by Wright et al. (2010) who found wood density was significantly correlated with growth and mortality. However, Rüger et al. (2012) showed that intrinsic growth rates, light response, and size response were all related to wood density, suggesting that wood density may represent a complex suite of trait interactions. Moreover, recent work applying engineering theory showed that greater strength for a given investment could be achieved by a larger diameter – which has questioned the ecological value of high wood density (Anten 2010; Larjavaara and Muller-Landau 2010). We did not find a correlation between growth or mortality and any of the other traits measured, suggesting that wood density may still be an important trait involved in the growth mortality trade-off. Future work should focus on the relationship among size, allocation, growth, and wood traits.

### Implications

A detailed understanding of the mechanisms allowing species coexistence in these diverse tropical forests is far from complete, but we finish by briefly discussing some potential implications of our results in light of the existing literature. Our results are not consistent with rank crossovers in the performance of species under different light conditions as a mechanism promoting species coexistence within this single functional group of shade-tolerant canopy species. Instead, we show a general (light independent) trade-off between growth and mortality that is consistent across the light gradient. In real terms, for a growth rate increase of 0.1 mm·mm^−1^·year^−1^, the annual mortality increases by 4.3%. Ultimately, species that have such higher mortality rates will need to produce more offspring to maintain their population growth rates, yet investment into offspring may trade off with characteristics at other ontogenic stages (Baraloto et al. 2005) such as seedling and sapling mortality rates. The long time-scale and novel size-standardized analysis we use provides new insights into seedling dynamics and indicate that even closely related species in one functional group have pronounced differences in growth and mortality strategies. To what degree these different strategies may help to explain the maintenance of coexistence in these highly diverse tropical forests is unclear. Nevertheless, understanding the role of light on species growth and mortality will allow us to address other potential niche axes more directly; especially as climate change alters resource and water availability.

In conclusion, our analysis of the growth and mortality of enrichment-planted seedlings of 15 species of dipterocarp over 8 years provides no clear support for species–specific responses to varying light conditions. Instead, our results support a general trade-off between growth and mortality across the light gradient from deeply shaded understory to large lighter gap conditions. This trade-off appears to be associated with wood density such that trees that have denser wood have lower diameter growth rates and lower mortality.
